# Distribution and antimicrobial resistance profiles of common blood culture isolates in a maternal and child health specialty hospital, Shenzhen (January 2021–October 2025), China

**DOI:** 10.3389/fmicb.2026.1756119

**Published:** 2026-03-06

**Authors:** Tongyan Ding, Shaoxiang Lin, Kaiyue Yang, Xiaojie Zhou, Shuyan Liu, Xiaochun Liu, Qiaoxin Zhang, Zhenwen Zhou

**Affiliations:** 1 Clinical Laboratory, Key Laboratory of Bacterial Antimicrobial Resistance and Prevention, Medical Research Institute of Maternal and Child, Longgang District Maternity & Child Healthcare Hospital of Shenzhen City (Affiliated Shenzhen Women and Children's Hospital (Longgang) of Shantou University Medical College), Shenzhen, Guangdong, China; 2 Clinical Laboratory, Guangzhou Women and Children's Medical Center, Guangzhou Medical University, Guangdong Provincial Clinical Research Center for Laboratory Medicine, Guangzhou, Guangdong, China; 3 The First Affiliated Hospital of Shantou University Medical College, Shantou, Guangdong, China

**Keywords:** antimicrobial resistance, blood culture, bloodstream infection, maternal and child health hospital, Shenzhen

## Abstract

**Objective:**

To investigate the distribution and antimicrobial resistance profiles of pathogens causing bloodstream infections (BSIs) in a maternal and child health hospital, providing evidence for rational clinical therapy.

**Methods:**

A retrospective analysis was conducted on 395 bacterial isolates recovered from positive blood cultures of inpatients at Longgang District Maternity & Child Healthcare Hospital of Shenzhen City between January 1, 2021 and October 31, 2025. Duplicate isolates from the same patient were excluded.

**Results:**

Among all isolates, Gram-positive bacteria accounted for 60.5% and Gram-negative bacteria for 39.5%. Coagulase-negative staphylococci (40.3%) were the most frequently detected, although many were likely contaminants. The major clinically relevant pathogens were *Escherichia coli* (28.6%), *Streptococcus agalactiae* (3.3%), *Klebsiella pneumoniae* subsp. pneumoniae (2.8%), and *Staphylococcus aureus* (2.3%). *Escherichia coli* showed a 44.3% Extended-Spectrum *β*-Lactamase (ESBL)-positive rate and elevated resistance to third-generation cephalosporins, with one carbapenem-resistant Enterobacteriaceae (CRE) isolate identified. *Klebsiella pneumoniae* exhibited overall low resistance levels. Among *Staphylococcus aureus*, methicillin-resistant *Staphylococcus aureus* (MRSA) accounted for 22.2%, all susceptible to vancomycin, linezolid, and tigecycline. *Streptococcus agalactiae* retained excellent *β*-lactam and glycopeptide susceptibility, whereas *Streptococcus pneumoniae* exhibited pronounced macrolide resistance.

**Conclusion:**

*Escherichia coli* and *Staphylococcus aureus* were the leading pathogens. The high ESBL rate in *Escherichia coli* and the detection of a CRE isolate indicate emerging *β*-lactam resistance risks. Although MRSA was detected, complete susceptibility to glycopeptides was preserved, highlighting the importance of ongoing monitoring.

## 1 Introduction

Bloodstream infection (BSI) is a severe, potentially life-threatening systemic infectious disease caused by the invasion of pathogenic microorganisms into the bloodstream, accompanied by the release of toxins and metabolic products (Deku et al., 2025; Holmes et al., 2025; Zhang et al., 2018). Although molecular biological techniques have recently been applied for the rapid detection of pathogens, blood culture remains the “gold standard” for diagnosing BSIs and sepsis, providing a reliable basis for definitive clinical diagnosis and rational antimicrobial therapy (Guo et al., 2024; Rapszky et al., 2025; Słabisz et al., 2024).

The spectrum of pathogens and antimicrobial resistance (AMR) patterns in BSIs are influenced by multiple factors, including region, hospital type, and patient characteristics such as age, immune status, and use of vascular catheters, leading to distinct differences among populations (Duse et al., 2021; Saavedra et al., 2023). For example, in general hospitals, BSIs are predominantly caused by Gram-negative bacteria, and multidrug-resistant (MDR) strains are frequently detected (Cui et al., 2024; Zhou et al., 2025). In contrast, in maternal and child health hospitals and neonatal intensive care units, Gram-positive bacteria such as coagulase-negative staphylococci are more commonly isolated, largely due to factors such as perinatal procedures, indwelling venous catheters, and immature immune function in this population (Xue et al., 2023; Zhang et al., 2022). Consequently, with the widespread use of antibiotics, improvements in obstetric and neonatal infection control, and changes in patient population structure, there may be a shift in the pathogen spectrum from Gram-positive to Gram-negative organisms (Bhutta et al., 2021).

Previous studies have demonstrated that inappropriate empirical antimicrobial therapy is an independent risk factor for poor prognosis in BSI patients, particularly among those infected with Enterobacteriaceae or other multidrug-resistant Gram-negative pathogens (Goldschmidt et al., 2025; von Beck et al., 2025). The occurrence of BSIs is also closely associated with various host-related factors, including physiological immune modulation during pregnancy and the puerperium, immature neonatal immunity, catheterization, and invasive perinatal procedures (Böll et al., 2020; Marco et al., 2025; Murt et al., 2023). Therefore, regional surveillance of pathogen distribution and antimicrobial resistance in maternal and neonatal populations is of great clinical importance for optimizing empirical antimicrobial therapy, improving early treatment outcomes, and curbing the spread of resistant organisms.

As a rapidly developing migrant megacity since China’s economic reform, Shenzhen has grown from a small town of tens of thousands in the early 1980s to over 17 million permanent residents. Its young and highly mobile population forms a distinctive demographic structure (Luo et al., 2025). Evidence suggests that the disease spectrum among local women and children has shifted over the past decade, reflecting the influence of ongoing demographic and environmental changes (He et al., 2019; Ren et al., 2015). However, despite this unique population background, epidemiological data on infectious diseases, particularly bloodstream infections, in maternal and child health care settings in Shenzhen remain limited.

To provide evidence-based guidance for rational antimicrobial use, this study retrospectively analyzed blood culture isolates collected from inpatients at Longgang District Maternity & Child Healthcare Hospital of Shenzhen City between January 1, 2021 and October 31, 2025, focusing on the distribution and resistance profiles of major pathogens. In summary, BSI remains a serious clinical infectious disease for which blood culture continues to be the most reliable diagnostic method. Given that antimicrobial resistance patterns vary across regions, strengthened local surveillance and the establishment of evidence-based treatment guidelines are essential. This study aims to systematically characterize the pathogen spectrum and resistance profiles of BSIs in a maternal and child health specialty hospital, providing epidemiological insights to support clinical management and antimicrobial stewardship.

## 2 Materials and methods

### 2.1 Bacterial strains

A total of 395 bacterial isolates were obtained from positive blood cultures of inpatients at Longgang District Maternity & Child Healthcare Hospital of Shenzhen City between January 1, 2021 and October 31, 2025. Duplicate isolates from the same patient were excluded from the analysis. This retrospective study analyzed de-identified clinical data obtained from routine blood culture testing and involved no patient contact, intervention, or additional sample collection. The study protocol was submitted to and approved by the Ethics Committee of the Longgang District Maternity & Child Healthcare Hospital of Shenzhen City (Approval No. LGFYKYXMLL-2025-67).

### 2.2 Methods

#### 2.2.1 Bacterial identification and antimicrobial susceptibility testing

Blood culture specimens were processed using the fully automated BD BACTEC FX blood culture system (Becton Dickinson and Company, USA). Bacterial identification and antimicrobial susceptibility testing (AST) were performed using the VITEK 2 Compact automated identification system (bioMérieux SA, Marcy-l’Étoile, France). For rare isolates or those that could not be reliably identified by the automated system, confirmation was conducted using the VITEK MS matrix-assisted laser desorption/ionization time-of-flight mass spectrometry (MALDI-TOF MS) system (bioMérieux SA, France). Interpretation of antimicrobial susceptibility results followed the Clinical and Laboratory Standards Institute (CLSI) Performance Standards for Antimicrobial Susceptibility Testing, 35th Edition (M100-Ed35, 2025). To avoid duplication, only the first positive blood culture yielding a specific pathogen from each patient was included in the analysis. In accordance with international recommendations, repeat isolates of the same species obtained within 30 days from the same patient were excluded unless they exhibited distinct antimicrobial susceptibility profiles or were recovered from different anatomical sites.

#### 2.2.2 Quality control strains

The quality control (QC) strains used in this study included *Escherichia coli* (ATCC 25922), *Klebsiella pneumoniae* (ATCC 700603), *Staphylococcus aureus* (ATCC 25923), *Pseudomonas aeruginosa* (ATCC 27853), *Enterococcus faecalis* (ATCC 29212), *Streptococcus pneumoniae* (ATCC 49619), and *Haemophilus influenzae* (ATCC 49247). The selection and testing of all QC strains were performed in accordance with the Clinical and Laboratory Standards Institute (CLSI) Performance Standards for Antimicrobial Susceptibility Testing, 35th Edition (M100-Ed35, 2025).

#### 2.2.3 Data dnalysis

Descriptive statistical methods were applied for data analysis. The distribution of bacterial species and antimicrobial resistance rates were expressed as frequencies and percentages. Results for bacterial species with small sample sizes were presented for reference only. Differences between different years were primarily presented descriptively.

## 3 Results

### 3.1 Bacterial distribution

#### 3.1.1 Major bacterial composition

A total of 395 bacterial isolates were obtained from positive blood cultures between January 2021 and October 2025. Among them, Gram-negative bacteria accounted for 39.5% (156/395), while Gram-positive bacteria accounted for 60.5% (239/395) (Table 1). Overall, the predominant isolates were coagulase-negative staphylococci and *Escherichia coli*, accounting for 40.3% (159/395) and 28.6% (113/395), respectively. Other frequently detected pathogens included *Klebsiella pneumoniae* subsp. pneumoniae (2.8%), *Staphylococcus aureus* (2.3%), *Streptococcus agalactiae* (3.3%), *Streptococcus pneumoniae* (2.0%), and *Enterococcus faecalis* (1.8%). In addition, rare isolates such as Salmonella sp. (1.3%) and *Pseudomonas aeruginosa* (1.0%) were also identified. Although a proportion of Coagulase-negative staphylococci isolates may represent contamination introduced during specimen collection, they were retained for subsequent antimicrobial resistance analyses due to their high detection frequency and clinical relevance in maternal-child healthcare settings.

**Table 1:** Bacterial composition of positive blood cultures between January 1, 2021 and October 31, 2025 (*n*, %).

Organism	2021 (*n* = 37)		2022 (*n* = 78)		2023 (*n* = 107)		2024 (*n* = 96)		2025 (*n* = 77)		Total (*n* = 395)	
	*n*	%	*n*	%	*n*	%	*n*	%	*n*	%	*n*	%
Gram-negative bacteria	24	64.9	25	32.1	34	31.8	51	53.1	32	41.6	156	39.5
*Escherichia coli*	11	29.7	21	26.9	23	21.5	34	35.4	24	31.2	113	28.6
*Klebsiella pneumoniae* subsp. pneumoniae	2	5.4	0	0.0	3	2.8	4	4.2	2	2.6	11	2.8
Salmonella sp.	1	2.7	1	1.3	1	0.9	1	1.0	1	1.3	5	1.3
*Pseudomonas aeruginosa*	2	5.4	0	0.0	0	0.0	0	0.0	2	2.6	4	1.0
Other Gram-negative bacteria	8	21.6	3	3.8	7	6.5	11	11.5	3	3.9	23	5.8
Gram-positive bacteria	13	35.1	53	67.9	73	68.2	45	46.9	45	58.4	239	60.5
Coagulase-negative staphylococci	4	10.8	38	48.7	60	56.1	27	28.1	29	37.7	159	40.3
*Streptococcus agalactiae*	4	10.8	2	2.6	1	0.9	5	5.2	1	1.3	13	3.3
*Staphylococcus aureus*	0	0.0	4	5.1	1	0.9	2	2.1	2	2.6	9	2.3
*Streptococcus pneumoniae*	1	2.7	2	2.6	1	0.9	4	4.2	1	1.3	8	2.0
*Enterococcus faecalis*	1	2.7	1	1.3	3	2.8	1	1.0	1	1.3	7	1.8
Other Gram-positive bacteria	3	8.1	6	7.7	7	6.5	9	9.4	11	14.3	43	10.9

In terms of annual trends, the species composition and relative proportions of positive blood culture isolates remained generally stable from 2021 to 2025, although some year-to-year fluctuations were observed. The detection rate of *Escherichia coli* varied between 29.7% in 2021 and 31.2% in 2025, remaining within the range of 20–35%. The proportion of *Klebsiella pneumoniae* subsp. pneumoniae ranged from 2.6 and 5.4%, while *Staphylococcus aureus* fluctuated at low levels (0.9–5.1%). The detection rates of *Streptococcus agalactiae* and *Streptococcus pneumoniae* remained relatively stable at approximately 1–5% of annual isolates. Rare pathogens, including *Pseudomonas aeruginosa* and Salmonella sp., consistently accounted for less than 3% of isolates throughout the study period.

Taken together, Gram-positive bacteria predominated among bloodstream isolates from 2021 to 2025. Although coagulase-negative staphylococci were detected most frequently, many were likely contaminants. Among clinically significant isolates, *Escherichia coli* was the most common pathogen, followed by *Klebsiella pneumoniae*, *Staphylococcus aureus*, and *Streptococcus agalactiae*, with the overall distribution remaining relatively stable over time.

#### 3.1.2 Distribution of major pathogens across clinical departments

In maternal and child populations, the spectrum of infectious pathogens exhibits distinct characteristics. *Escherichia coli* is the most common Gram-negative pathogen associated with infections in obstetric and neonatal settings, whereas *Staphylococcus aureus*—particularly methicillin-resistant *Staphylococcus aureus* (MRSA)—is frequently implicated in neonatal skin and catheter-related infections and serves as a key focus of hospital surveillance. Although *Streptococcus agalactiae* can cause neonatal sepsis, its detection rate remains relatively low and antimicrobial resistance is limited. Based on these observations, this study primarily analyzed the distribution patterns of *Escherichia coli* and *Staphylococcus aureus*.

According to Table 2, isolates of *Escherichia coli* and *Staphylococcus aureus* were predominantly recovered from the neonatal unit, obstetrics, and pediatrics departments. A total of 113 *Escherichia coli* isolates were identified, mainly from obstetrics (47 isolates, 41.6%) and the neonatal unit (46 isolates, 40.7%), followed by pediatrics (13 isolates, 11.5%); gynecology and other departments accounted for 5.3 and 0.9%, respectively. A total of 9 *Staphylococcus aureus* isolates were detected, with the highest proportion originating from the neonatal unit (6 isolates, 66.7%), followed by pediatrics (2 isolates, 22.2%); isolates from obstetrics and other departments each accounted for 11.1%, while no isolates were identified in gynecology.

**Table 2:** Departmental distribution of major pathogenic bacteria (*n*, %).

Departments	*Escherichia coli*		*Staphylococcus aureus*	
	*n*	%	*n*	%
Neonatal unit	46	40.7	6	66.7
Pediatrics	13	11.5	2	22.2
Obstetrics	47	41.6	1	11.1
Gynecology	6	5.3	0	0
Other	1	0.9	0	0

#### 3.1.3 Distribution of major pathogens by sex in the neonatal unit and pediatrics departments

**Figure 1:**
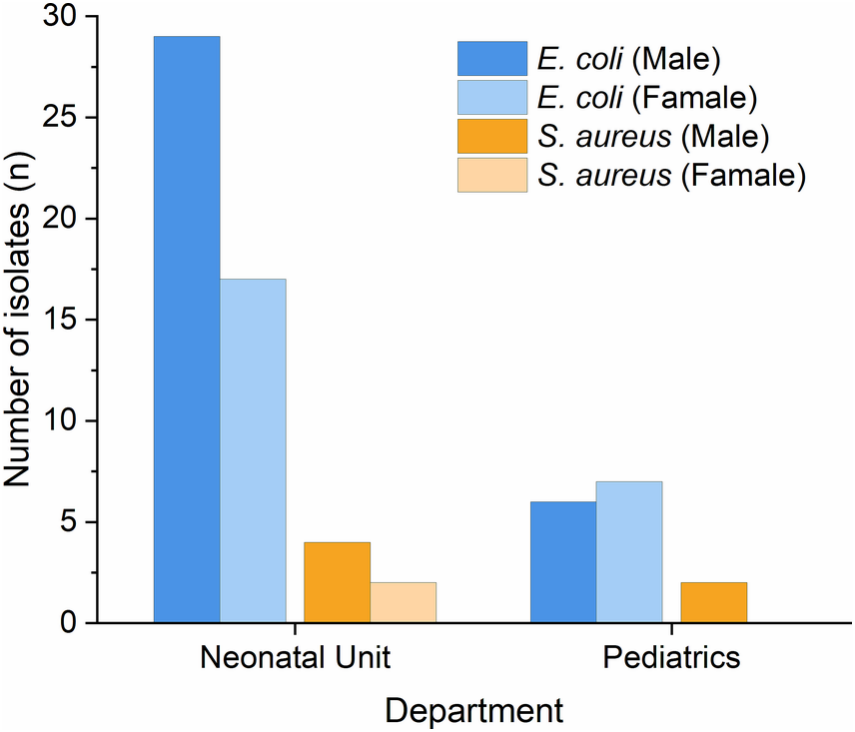
Sex distribution of major pathogenic bacteria. *Escherichia coli*: *E. coli*; *Staphylococcus aureus*: *S. aureus*. Grouped by department and sex.

Both *Escherichia coli* and *Staphylococcus aureus* isolates from the neonatal unit and pediatrics departments showed certain sex-related differences (Figure 1). A total of 59 *Escherichia coli* isolates were identified, including 29 (49.1%) from male neonates and 17 (28.8%) from female neonates in the neonatal department, as well as 7 (11.9%) from male children and 6 (10.2%) from female children in the pediatrics department. A total of 8 *Staphylococcus aureus* isolates were detected, comprising 4 (50.0%) from male neonates and 2 (25.0%) from female neonates in the neonatal department, and 2 (25.0%) from male children in the pediatrics department; no isolates were found in female children. Overall, both pathogens were most frequently isolated from the neonatal department, with a higher number of isolates detected in male neonates compared to female neonates.

### 3.2 Antimicrobial resistance of major gram-negative bacteria

#### 3.2.1 Escherichia coli

A total of 113 *Escherichia coli* isolates were obtained, among which 50 (44.3%) were extended-spectrum *β*-lactamase (ESBL) producers. No significant temporal trend was observed in the proportion of ESBL-producing *Escherichia coli* from 2021 to 2024 (χ^2^ test for trend, *p* = 0.35). The antimicrobial susceptibility profiles are summarized in Table 3. Among *β*-lactam/*β*-lactamase inhibitor combinations, resistance to amoxicillin-clavulanate (4.0%) and piperacillin-tazobactam (4.4%) remained low, with piperacillin-tazobactam showing a susceptibility rate exceeding 94%. Resistance to cephalosporins was markedly higher, particularly cefuroxime sodium (48%), cefuroxime axetil (48%), and ceftriaxone (44.2%), whereas lower resistance was observed for ceftazidime (12.4%) and cefoxitin (2.7%). Cefepime showed moderate resistance at 18.6%.

**Table 3:** Antimicrobial resistance profile of *Escherichia coli* to commonly used antibiotics.

Antimicrobial agents	Resistance rate (%)	Susceptibility rate (%)	Intermediate rate (%)
Amikacin	1.8	98.2	0.0
Amoxicillin-clavulanate	4.0	81.3	14.7
Ertapenem	0.8	98.2	1.0
Trimethoprim/sulfamethoxazole	52.2	47.8	0
Piperacillin-tazobactam	4.4	94.7	0.9
Tigecycline	0	100	0
Cefepime	18.6	75.2	6.2
Cefuroxime sodium	48	52	0
Cefuroxime axetil	48	52	0
Cefoperazone-sulbactam	2.7	93.3	4
Ceftriaxone	44.2	55.8	0
Ceftazidime	12.4	82.3	5.3
Cefoxitin	2.7	97.3	0
Imipenem	0.9	97.4	1.7
Levofloxacin	35.4	16.8	47.8

Intermediate (I) susceptibility to levofloxacin in *Escherichia coli* may retain potential clinical relevance under appropriate exposure conditions and should be interpreted with caution, in accordance with CLSI interpretive criteria.

Carbapenem resistance was rare, with only one carbapenem-resistant Enterobacteriaceae (CRE) isolate identified. Tigecycline, cefoperazone–sulbactam, and amikacin remained largely active, whereas non-*β*-lactam agents such as fluoroquinolones (high resistance with a substantial intermediate proportion) and trimethoprim/sulfamethoxazole showed reduced activity in this dataset.

In summary, *Escherichia coli* isolates displayed high susceptibility to carbapenems, aminoglycosides, tigecycline, and piperacillin-tazobactam, while demonstrating notable resistance to third-generation cephalosporins, fluoroquinolones, and trimethoprim/sulfamethoxazole. The detection of a single CRE isolate underscores the need for continuous surveillance and prudent antimicrobial stewardship.

#### 3.2.2 *Klebsiella pneumoniae* subsp. pneumoniae

A total of 11 *Klebsiella pneumoniae* subsp. pneumoniae isolates were identified, and the antimicrobial susceptibility patterns are summarized in Table 4. All isolates were fully susceptible (100%) to amikacin, amoxicillin-clavulanate, piperacillin-tazobactam, ertapenem, imipenem, tigecycline, and cefoperazone-sulbactam. In contrast, trimethoprim/sulfamethoxazole showed a resistance rate of 9.1% (90.9% susceptible). Lower susceptibility was observed for levofloxacin (36.4% resistant and 18.2% intermediate) and for third-generation cephalosporins, with resistance rates of 36.4% for ceftriaxone and 27.3% for ceftazidime, whereas cefoxitin remained fully active (0% resistance).

**Table 4:** Antimicrobial resistance profile of *Klebsiella pneumoniae* to commonly used antibiotics.

Antimicrobial agents	Resistance rate (%)	Susceptibility rate (%)	Intermediate rate (%)
Amikacin	0	100	0
Amoxicillin-clavulanate	0	100	0
Ertapenem	0	100	0
Trimethoprim/sulfamethoxazole	9.1	90.9	0
Piperacillin-tazobactam	0	100	0
Tigecycline	0	100	0
Cefoperazone-sulbactam	0	100	0
Ceftriaxone	36.4	63.6	0
Ceftazidime	27.3	72.7	0
Cefoxitin	0	100	0
Imipenem	0	100	0
Levofloxacin	36.4	45.4	18.2

For certain bacterial species with small sample sizes, resistance rates are provided for reference only.

In summary, *Klebsiella pneumoniae* subsp. pneumoniae isolates demonstrated overall low antimicrobial resistance, with high susceptibility to carbapenems, aminoglycosides, tigecycline, and *β*-lactam/*β*-lactamase inhibitor combinations. However, resistance to third-generation cephalosporins and fluoroquinolones was present in a proportion of isolates, underscoring the continued need for targeted surveillance.

#### 3.2.3 Salmonella sp. and *pseudomonas aeruginosa*

A total of five Salmonella isolates and four *Pseudomonas aeruginosa* isolates were obtained. All Salmonella isolates were fully susceptible (100%) to *β*-lactam/*β*-lactamase inhibitor combinations, carbapenems, cephalosporins (except ceftazidime), aminoglycosides, tigecycline, and trimethoprim/sulfamethoxazole. Resistance to ceftazidime was detected in one isolate (20%), and 40% of isolates showed intermediate susceptibility to levofloxacin.

For *Pseudomonas aeruginosa*, complete susceptibility (100%) was observed to aminoglycosides (amikacin, gentamicin) and fluoroquinolones (ciprofloxacin, levofloxacin). In contrast, resistance emerged to multiple *β*-lactam and carbapenem agents, including cefepime (100%), cefoperazone-sulbactam (50%), ceftazidime (25%), meropenem (100%), and imipenem (25%). Tobramycin resistance was 25%, while colistin remained fully active.

In summary, Salmonella isolates showed overall high susceptibility to the major clinically used antimicrobial agents, with only limited resistance observed to ceftazidime and intermediate susceptibility to levofloxacin. *Pseudomonas aeruginosa* isolates demonstrated preserved susceptibility to aminoglycosides and fluoroquinolones, while resistance to several *β*-lactam agents and carbapenems was detected.

### 3.3 Antimicrobial resistance of major gram-positive bacteria

#### 3.3.1 Coagulase-negative aphylococci

A total of 159 coagulase-negative staphylococci isolates were obtained. The antimicrobial susceptibility profiles are presented in Table 5. Overall, the isolates exhibited variable resistance patterns across different classes of antibiotics. Among non–β-lactam agents, the highest resistance rate was observed for erythromycin (57.9%), followed by clindamycin (22.2%) and trimethoprim/sulfamethoxazole (8.9%). Rifampin and gentamicin demonstrated low resistance rates of 1.9 and 2.5%, respectively, with susceptibility rates exceeding 90% for both agents. Regarding β-lactam antibiotics, cefoxitin showed a resistance rate of 52.4%, indicating a substantial proportion of methicillin-resistant coagulase-negative staphylococci. In contrast, vancomycin and teicoplanin maintained excellent antimicrobial activity, with susceptibility rates of 100 and 99.0%, respectively. For fluoroquinolones, levofloxacin exhibited a resistance rate of 11.4%, with an additional 3.2% showing intermediate susceptibility.

**Table 5:** Antimicrobial resistance profile of coagulase-negative staphylococci to commonly used antibiotics.

Antimicrobial agents	Resistance rate (%)	Susceptibility rate (%)	Intermediate rate (%)
Trimethoprim/Sulfamethoxazole	8.9	91.1	0
Erythromycin	57.9	39.6	1.9
Clindamycin	22.2	70.6	7.2
Rifampin	1.9	97.5	0.6
Gentamicin	2.5	90.5	7.0
Teicoplanin	0	99.0	1.0
Cefoxitin	52.4	46.9	0
Vancomycin	0	100	0
Levofloxacin	11.4	85.4	3.2

Overall, coagulase-negative staphylococci displayed high susceptibility to glycopeptides and rifampin, while moderate to high resistance was observed to macrolides, clindamycin, and cefoxitin.

#### 3.3.2 Streptococcus agalactiae

A total of 13 *Streptococcus agalactiae* isolates were obtained. Antimicrobial susceptibility testing showed that all isolates were fully susceptible to ampicillin, penicillin, vancomycin, linezolid, and tigecycline (Table 6). Low-level resistance was observed among fluoroquinolones, with resistance rates of 7.7% for both levofloxacin and moxifloxacin. Overall, *Streptococcus agalactiae* remained highly susceptible to major clinically used antimicrobial agents, with a generally low level of resistance.

**Table 6:** Antimicrobial resistance profile of *Streptococcus agalactiae* to commonly used antibiotics.

Antimicrobial agents	Resistance rate (%)	Susceptibility rate (%)	Intermediate rate (%)
Ampicillin	0	100	0
Linezolid	0	100	0
Moxifloxacin	7.7	92.3	0
Penicillin	0	100	0
Tigecycline	0	100	0
Vancomycin	0	100	0
Levofloxacin	7.7	92.3	0

For certain bacterial species with small sample sizes, resistance rates are provided for reference only.

#### 3.3.3 Staphylococcus aureus

A total of nine *Staphylococcus aureus* isolates were obtained, and their antimicrobial susceptibility profiles are shown in Table 7. All isolates were resistant to penicillin, and 22.2% were resistant to oxacillin, indicating the presence of methicillin-resistant *Staphylococcus aureus* (MRSA). Vancomycin, teicoplanin, tigecycline, linezolid, and daptomycin showed 100% susceptibility. Among macrolide–lincosamide agents, erythromycin resistance was 33.3% with 11.1% intermediate susceptibility, while clindamycin resistance was 22.2%. Fluoroquinolone activity remained favorable, with 88.9% susceptibility to moxifloxacin and 11.1% resistance to levofloxacin. Gentamicin, trimethoprim/sulfamethoxazole, and rifampin each showed a resistance rate of 11.1%.

**Table 7:** Antimicrobial resistance profile of *Staphylococcus aureus* to commonly used antibiotics.

Antimicrobial agents	Resistance rate (%)	Susceptibility rate (%)	Intermediate rate (%)
Oxacillin	22.2	77.8	0
Daptomycin	0	100	0
Trimethoprim/sulfamethoxazole	11.1	88.9	0
Erythromycin	33.3	55.6	11.1
Clindamycin	22.2	77.8	0
Rifampin	11.1	88.9	0
Linezolid	0	100	0
Moxifloxacin	0	88.9	0
Penicillin	100	0	0
Gentamicin	11.1	88.9	0
Tigecycline	0	100	0
Teicoplanin	0	100	0
Vancomycin	0	100	0
Levofloxacin	11.1	88.9	0

For certain bacterial species with small sample sizes, resistance rates are provided for reference only.

Overall, *Staphylococcus aureus* remained susceptible to most clinically used agents, although resistance to *β*-lactams and macrolide–lincosamide antibiotics was evident. The proportion of MRSA was 22.2%.

#### 3.3.4 Streptococcus pneumoniae

A total of eight *Streptococcus pneumoniae* isolates were obtained, and their antimicrobial susceptibility profiles are shown in Table 8. Linezolid, chloramphenicol, moxifloxacin, telithromycin, ceftriaxone (meningitis regimen), vancomycin, and ofloxacin demonstrated 100% susceptibility, with no resistant or intermediate isolates detected. In contrast, erythromycin showed a 100% resistance rate, representing the highest level of resistance among all tested agents. Tetracycline also exhibited a high resistance rate of 85.7%, with only 14.3% of isolates remaining susceptible. Trimethoprim/sulfamethoxazole displayed a resistance rate of 44.4% and an intermediate rate of 33.3%, indicating reduced activity. For meropenem, 66.7% of isolates were susceptible and 33.3% showed intermediate susceptibility.

**Table 8:** Antimicrobial resistance profile of *Streptococcus pneumoniae* to commonly used antibiotics.

Antimicrobial agents	Resistance rate (%)	Susceptibility rate (%)	Intermediate rate (%)
Trimethoprim/sulfamethoxazole	44.4	22.2	33.3
Erythromycin	100	0	0
Linezolid	0	100	0
Chloramphenicol	0	100	0
Meropenem	0	66.7	33.3
Moxifloxacin	0	100	0
Tetracycline	85.7	14.3	0
Telithromycin	0	100	0
Ceftriaxone (meningitis)	0	100	0
Vancomycin	0	100	0
Ofloxacin	0	100	0

For certain bacterial species with small sample sizes, resistance rates are provided for reference only.

In summary, *Streptococcus pneumoniae* isolates maintained excellent susceptibility to *β*-lactams used in meningitis regimens, glycopeptides, oxazolidinones, and fluoroquinolones, whereas notable resistance was observed to erythromycin, tetracycline, and trimethoprim/sulfamethoxazole.

#### 3.3.5 Enterococcus faecalis

A total of seven *Enterococcus faecalis* isolates were obtained, and their antimicrobial susceptibility profiles are summarized in Table 9. The isolates showed uniformly high susceptibility to *β*-lactam antibiotics, with ampicillin and penicillin both demonstrating 100% susceptibility. Glycopeptides (vancomycin and teicoplanin), oxazolidinones (linezolid), and tigecycline likewise exhibited complete activity against all isolates.

**Table 9:** Antimicrobial resistance profile of *Enterococcus faecalis* to commonly used antibiotics.

Antimicrobial agents	Resistance rate (%)	Susceptibility rate (%)	Intermediate rate (%)
Ampicillin	0	100	0
High-level gentamicin	28.6	71.4	0
Erythromycin	42.9	14.2	42.9
Linezolid	0	100	0
Penicillin	0	100	0
Tigecycline	0	100	0
Teicoplanin	0	100	0
Vancomycin	0	100	0
Levofloxacin	0	85.7	14.3

For certain bacterial species with small sample sizes, resistance rates are provided for reference only.

High-level gentamicin resistance was observed in 28.6% of isolates, indicating the presence of high-level aminoglycoside resistance in a subset of strains. For macrolides, erythromycin showed the highest resistance, with a resistance rate of 42.9% and an additional 42.9% demonstrating intermediate susceptibility, leaving only 14.2% fully susceptible. Levofloxacin exhibited favorable activity, with no resistant isolates detected and 85.7% susceptibility.

Overall, *Enterococcus faecalis* remained highly susceptible to *β*-lactams, glycopeptides, oxazolidinones, and tigecycline, whereas reduced susceptibility was noted for erythromycin and high-level gentamicin.

## 4 Discussion

Blood culture is universally recognized as the gold standard for the diagnosis of bloodstream infection (BSI) because it enables the recovery of viable pathogens and the performance of conventional antimicrobial susceptibility testing (Reszetnik et al., 2024). Therefore, it plays a pivotal role in guiding empirical therapy, optimizing targeted treatment, and controlling hospital outbreaks (Khanal et al., 2025). Although rapid molecular assays (such as multiplex PCR) can identify probable pathogens in a shorter time, these methods still cannot directly provide phenotypic susceptibility data, and their sensitivity is not yet sufficient to fully replace traditional blood cultures. Thus, clinicians must continue to rely on blood culture results to determine definitive antimicrobial regimens (Hattab et al., 2024). A positive blood culture indicates that live bacteria have breached host barriers and entered the bloodstream, providing direct evidence of severe systemic infection. This finding is closely associated with disease progression, prognosis, and mortality, and it is particularly critical in patients with severe sepsis or septic shock who require early, precise antimicrobial therapy (Ong et al., 2024). The clinical significance of blood culture is even more pronounced in highly vulnerable populations-such as neonates, pregnant and puerperal women, and immunocompromised patients-because the definitive diagnosis of neonatal sepsis still depends on blood culture, and a positive result directly affects the selection and duration of antimicrobial therapy (Gad et al., 2024; Kariniotaki et al., 2024).

During the period between January 1, 2021 and October 31, 2025, the overall blood culture positivity rate in our hospital was relatively low. A total of 395 bacterial isolates were recovered, of which Gram-positive bacteria accounted for 60.5% (239/395) and Gram-negative bacteria for 39.5% (156/395). The most frequently isolated organisms were coagulase-negative staphylococci (40.3%) and *Escherichia coli* (28.6%), followed by *Klebsiella pneumoniae* subsp. pneumoniae (2.8%), *Streptococcus agalactiae* (3.3%), *Staphylococcus aureus* (2.3%), *Streptococcus pneumoniae* (2.0%), and *Enterococcus faecalis* (1.8%). In addition, rare pathogens such as Salmonella sp. (1.3%) and *Pseudomonas aeruginosa* (1.0%) were also detected. The overall pathogen spectrum was generally consistent with surveillance data from maternal and child or neonatal specialty hospitals in China, indicating that BSIs in this population remain predominantly caused by Gram-positive organisms, although the proportion of Gram-negative bacteria shows an upward trend (Zhang et al., 2022; Zhou et al., 2023). Given the small number of isolates for certain pathogens (n < 10), the corresponding resistance rates may be influenced by random variation and should be interpreted with caution. It should be emphasized that, although Coagulase-negative staphylococci had the highest isolation rate, these organisms are common skin colonizers, and their recovery from blood cultures is often difficult to distinguish as true bacteremia versus contamination (Dargere et al., 2018; Rybak et al., 2025). Because this study was retrospective and based solely on laboratory data, complete clinical information (such as fever status, inflammatory markers, or repeat positive cultures) was not available to determine the clinical significance of the isolates. Therefore, coagulase-negative staphylococci were not included in the analysis of major pathogens. Nevertheless, the antimicrobial resistance profiles can still serve as important background information in a maternal and child healthcare setting. These profiles may reflect local antibiotic selection pressure, catheter or device-related colonization risk, and the antimicrobial resistance ecology of neonatal and obstetric wards.

Our data further indicated that *Escherichia coli* and *Staphylococcus aureus* were the major pathogens, mainly isolated from the neonatal and obstetrics departments, which is closely associated with the patient composition and clinical characteristics of a maternal and child healthcare hospital. Among them, *Escherichia coli* was predominantly derived from the neonatal department (44.7%) and obstetrics department (34.0%), while *Staphylococcus aureus* was mainly isolated from the neonatal (55.6%) and pediatrics (22.2%) departments. Neonates are more susceptible to opportunistic infections because of their immature immune system, inadequate skin barrier function, and frequent exposure to invasive procedures (such as venous catheterization and blood sampling). Multicenter studies in China have similarly reported that *Escherichia coli* and *Staphylococcus aureus* are among the leading causative agents of neonatal BSIs. Yin et al. (2024) found that *Escherichia coli* had the highest detection rate in early-onset sepsis (27.2%), whereas *Staphylococcus aureus* accounted for up to 41.5% of community-acquired late-onset sepsis, underscoring its importance as a neonatal BSI pathogen. These findings are consistent with the distribution pattern observed in our study and suggest that both organisms are major causes of neonatal bacteremia.

With respect to sex distribution, we observed that both *Escherichia coli* and *Staphylococcus aureus* showed a male predominance among neonates. In our cohort, male neonates accounted for 49.1% of *Escherichia coli*–positive cases and female neonates for 28.8%; for *Staphylococcus aureus*, 50.0% of positive cases were in male neonates and 25.0% in female neonates. Similar trends have been reported previously. Migliori et al. (2023) pointed out that male neonates tend to be disadvantaged in several neonatal complications and have higher susceptibility to infections, suggesting that sex-related differences may play a role in the occurrence of neonatal infections. This may be associated with relatively delayed immune maturation in males, androgen-mediated suppression of immune cell activity, and sex-related genetic or epigenetic regulatory mechanisms.

In this study, *Escherichia coli* remained the predominant Gram-negative pathogen, with an antimicrobial resistance profile largely consistent with national surveillance; however, the ESBL rate reached 44.3%, suggesting that peripartum antibiotic exposure and intestinal microbiota alterations may contribute to its prevalence in maternal-child populations. The detection of a single CRE isolate is of particular concern, as CRE colonization in neonatal units carries a high risk of onward transmission, underscoring the need for active CRE screening and surveillance of key resistance determinants such as blaKPC and blaNDM (Yin et al., 2021). In contrast, *Klebsiella pneumoniae* subsp. pneumoniae exhibited generally low resistance, although emerging resistance to third-generation cephalosporins and fluoroquinolones was observed, consistent with trends reported in pediatric settings nationwide. Although no carbapenem-resistant isolates were detected, the occurrence of CRKP outbreaks in NICU and obstetric wards reported elsewhere highlights the importance of continued vigilance (Zhang et al., 2022). Among non-Enterobacterales, Salmonella remained broadly susceptible, whereas *Pseudomonas aeruginosa* demonstrated resistance to multiple *β*-lactams and carbapenems, a pattern potentially associated with efflux pump overexpression or OprD loss. Overall, the findings indicate an increasing burden of Gram-negative resistance in maternal–child healthcare settings, characterized by a substantial prevalence of ESBL-producing strains and the early emergence of CRE. These observations emphasize the need to strengthen antimicrobial stewardship, resistance gene surveillance, and targeted infection control strategies for neonates and pregnant or postpartum women. Molecular confirmation of resistance genes (e.g., bla_CTX-M, bla_KPC, bla_NDM) was not performed in this study, and the related conclusions are therefore based solely on phenotypic findings. Future integration of resistance gene detection and molecular epidemiological analysis into routine surveillance could enable precise identification and tracing of transmission pathways of resistant organisms, thereby improving the specificity of infection control measures and strengthening early warning and rapid response to antimicrobial resistance outbreaks.

From a maternal-child healthcare perspective, the distribution of major pathogens differs across patient populations, corresponding to distinct resistance risk profiles. *Escherichia coli* was more prevalent in neonates and obstetric patients than in pediatric patients, indicating that Gram-negative resistance risks, such as ESBL production, warrant particular attention in peripartum- and neonatal-related bloodstream infections. In contrast, *Staphylococcus aureus* was more frequently identified among neonates, highlighting the need to also consider Gram-positive resistance risks and infection prevention in this population. Overall, stratified interpretation of antimicrobial resistance surveillance data based on patient populations may help optimize empirical treatment decision-making and antimicrobial stewardship in maternal-child healthcare settings; moreover, local resistance profiles can inform the selection of empirical antibiotic regimens and support early adjustment following susceptibility testing results.

Among Gram-positive pathogens, coagulase-negative staphylococci were the predominant isolates and exhibited high resistance to macrolides and cefoxitin, indicating a considerable burden of methicillin-resistant coagulase-negative staphylococci in the hospital setting. Despite this, susceptibility to glycopeptides and rifampin remained largely preserved. *Staphylococcus aureus* was detected infrequently, and 22.2% of the isolates were identified as MRSA, a proportion slightly lower than that reported in several pediatric centers (La Vecchia et al., 2022). All *Staphylococcus aureus* isolates were fully susceptible to vancomycin, linezolid, and tigecycline, suggesting that glycopeptides and oxazolidinones continue to provide reliable therapeutic coverage for MRSA infections. *Streptococcus pneumoniae* showed pronounced macrolide and tetracycline resistance, in line with multicenter neonatal surveillance indicating persistently high macrolide resistance (Zhang et al., 2022), likely driven by long-term antibiotic selection pressure. Nevertheless, *Streptococcus pneumoniae* remained fully susceptible to meningitis-dose ceftriaxone and glycopeptides. *Enterococcus faecalis* also demonstrated broad susceptibility to major first-line antimicrobial agents, with high-level gentamicin resistance being the principal concern when considering synergistic regimens. Overall, these findings highlight the importance of optimizing antibiotic use, particularly macrolides, and reinforce the need for continued surveillance of MRSA in maternal and child healthcare settings.

This study has several limitations. First, as a single-center, retrospective, laboratory-based study, the number of isolates in some years (e.g., 2025, which was only counted through October 31) was relatively small, which may have resulted in random fluctuations in pathogen distribution and resistance rates. To minimize this bias, we combined nearly 5 years of data to reflect the overall trend. Second, due to the lack of complete clinical information, we were unable to further assess the correlation between isolated strains and true clinical infection. Third, infection prevention and control measures and antimicrobial stewardship interventions implemented during the study period may have influenced the dynamics of pathogen composition and antimicrobial resistance. It should also be noted that for some infrequently isolated species (such as *Klebsiella pneumoniae* and *Pseudomonas aeruginosa*), the sample size was small, and their resistance rates should therefore be interpreted with caution. Future multicenter studies with larger sample sizes, complemented by molecular epidemiological approaches, are needed to further validate and extend the present findings.

## 5 Conclusion

The pathogens responsible for severe sepsis and their antimicrobial resistance profiles are constantly evolving. Continuous surveillance of pathogen distribution and resistance trends in blood cultures provides valuable insights into the epidemiological characteristics of bloodstream infections in maternal and child healthcare settings, which is crucial for guiding empirical antimicrobial therapy in this population. Due to pregnancy-associated immune modulation and the immature immune systems of neonates, individuals in this group are more vulnerable to pathogen invasion, often experiencing rapid disease progression and poor prognosis. The resistance patterns identified in this study offer practical guidance for optimizing clinical treatment regimens and provide a scientific basis for antimicrobial stewardship and infection control strategies in maternal and child healthcare institutions.

## Data Availability

The original contributions presented in the study are included in the article/supplementary material, further inquiries can be directed to the corresponding authors.
